# Segmentation of Anatomical Structures of the Left Heart from Echocardiographic Images Using Deep Learning

**DOI:** 10.3390/diagnostics13101683

**Published:** 2023-05-09

**Authors:** MHD Jafar Mortada, Selene Tomassini, Haidar Anbar, Micaela Morettini, Laura Burattini, Agnese Sbrollini

**Affiliations:** Department of Information Engineering, Università Politecnica delle Marche, 60121 Ancona, Italy; s1101958@studenti.univpm.it (M.J.M.); s.tomassini@pm.univpm.it (S.T.); s1101956@studenti.univpm.it (H.A.); m.morettini@staff.univpm.it (M.M.); a.sbrollini@staff.univpm.it (A.S.)

**Keywords:** left heart segmentation, echocardiography, YOLOv7, deep learning, convolutional neural networks, U-Net

## Abstract

Knowledge about the anatomical structures of the left heart, specifically the atrium (LA) and ventricle (i.e., endocardium—Vendo—and epicardium—LVepi) is essential for the evaluation of cardiac functionality. Manual segmentation of cardiac structures from echocardiography is the baseline reference, but results are user-dependent and time-consuming. With the aim of supporting clinical practice, this paper presents a new deep-learning (DL)-based tool for segmenting anatomical structures of the left heart from echocardiographic images. Specifically, it was designed as a combination of two convolutional neural networks, the YOLOv7 algorithm and a U-Net, and it aims to automatically segment an echocardiographic image into LVendo, LVepi and LA. The DL-based tool was trained and tested on the Cardiac Acquisitions for Multi-Structure Ultrasound Segmentation (CAMUS) dataset of the University Hospital of St. Etienne, which consists of echocardiographic images from 450 patients. For each patient, apical two- and four-chamber views at end-systole and end-diastole were acquired and annotated by clinicians. Globally, our DL-based tool was able to segment LVendo, LVepi and LA, providing Dice similarity coefficients equal to 92.63%, 85.59%, and 87.57%, respectively. In conclusion, the presented DL-based tool proved to be reliable in automatically segmenting the anatomical structures of the left heart and supporting the cardiological clinical practice.

## 1. Introduction

Echocardiography is a non-invasive medical technique able to acquire images of the heart; it can be used to evaluate cardiac structure and function. Echocardiographic images are frames of a video usually acquired during all phases of the cardiac cycle. The frames with the highest information are the end-systolic (ES) and the end-diastolic (ED) frames. The echocardiographic exam is still manually performed by clinicians who optimize the image acquisition, detect the cardiac chambers and segment the anatomical structures. In practice, they move an echocardiographic probe on the patient’s chest to optimize visualization, and consequently, variations in imaging accuracy arise. When the quality of the image is sufficiently strong, clinicians move a pointer on the echocardiographic screen and manually segment and measure dimensions of cardiac anatomical structures. Thus, echocardiography is still user-dependent and subjective [1]. In many clinical applications, echocardiographic image segmentation is a crucial step [2]. For example, it allows the measurement of the myocardial thickness in the case of myocardial ischemia [3], the estimation of valve area in the case of ventricular stenosis [4] or the quantification of ventricular volume during the cardiac cycle to assess the ejection fraction in the case of heart failure [5].

To reduce the subjectiveness of echocardiography and to support clinicians in cardiac structure segmentation, the use of automatic algorithms as decision support systems is desirable. However, getting reliable ones remains challenging. Indeed, ultrasound images are usually characterized by a low signal-to-noise ratio [6], location and dimensions of anatomical structures may act as confounders due to intrasubject variability, and the application of conventional image processing methods (e.g., edge detection and shape models) may face many technical issues [7], such as the inference of physical properties from pixel intensity. Moreover, an echocardiography test is usually composed of a sequence of images, frames of a video, that represent all phases of a cardiac cycle; thus, automatic processing of ultrasound images should be fast and able to deal with a high amount of data.

Deep learning (DL) methods may appear as efficient alternatives to conventional image processing methods [8,9,10]. In particular, convolutional neural networks (CNNs) are powerful tools able to automatically learn and extract relevant features from the input images [11]. Thus, in the context of echocardiography, the DL-method may support identification and segmentation of the main anatomical structures of the left heart. Thus, the aim of the present work is to present a new DL-based tool to identify and segment the most important anatomical structures of the left heart, namely, the left ventricular endocardium, the left ventricular epicardium and the left atrium.

## 2. Related Works

In the literature, nine papers [12,13,14,15,16,17,18,19,20] report the application of DL to segment echocardiography images. Leclerc et al. (2019) [13] compared multiple DL methods for left ventricular endocardium and myocardium segmentation and demonstrated the superiority of encoder–decoder-based architectures in relation to state-of-the-art non-DL methods. Moradi et al. (2019) [14] used the U-Net to segment the left ventricle by improving the U-Net architecture in MFP-U-Net. This new CNN had extra convolution layers for performing feature maps and improving the left ventricular segmentation performance. Kim et al. [15] aimed to segment the left ventricular endocardium and left ventricular myocardium. They designed algorithms considering porcine images and tested it on human images. Despite the adequate performance, the main limitation was related to the fact that the designed method was performed upon open-chest pigs, a technique which has better quality than human echocardiographic images. Girum et al. [16] combined a modified U-Net architecture with an FCN encoder in order to improve feature extraction and allow the system to learn from its own mistakes. Liu et al. [12] used a bilateral segmentation network to extract deep features and a pyramid local-attention algorithm to enhance features within compact and sparse neighboring contexts. Lei et al. [17] proposed Cardiac-SegNet, a system combining a U-Net (performing feature extraction), a fully convolutional single-state object detector (segmenting the image into the region of interest) and a mask head network (performing segmentation). Alam et al. [18] proposed a two-parallel pipeline for ES frame and ED frame segmentation by using DeepResU-Net. Distinct from the others, Saeed et al. [19] used self-supervised algorithms (DeepLapV3, SimCLR, BOYL and U-Net) to segment the left ventricle in order to overcame the lack of labeled data. Finally, Zhuang et al. (2021) [20] used an object-detection method, the YOLOv3 algorithm, to detect three points of ventricular chamber and segment the ventricles. Despite the innovativeness of the methods and their high performance, all these studies focused on segmenting the ventricle but not all its anatomical structures.

Comparison with the literature shows that the main innovative aspects of our DL-based tool are: (1) it integrates the YOLOv7 algorithm as a module for chamber identification and a module for chamber segmentation by U-Net, supporting the clinicians in all phases of the echocardiographic exam, (2) it is able to segment three important anatomical structures of the left heart simultaneously, and (3) it is implemented in a cloud-computing environment, allowing the method to be easily-reproducible and machine-independent.

## 3. Materials and Methods

### 3.1. Data

The CAMUS dataset [9] was published in 2019 and included echocardiographic images acquired from 500 patients at University Hospital of St Etienne in France. The images were acquired by a Vivid E95 ultrasound scanner (from GE Vingmed Ultrasound) with a GE M5S probe (General Electrics Healthcare, Chicago, IL, USA). This dataset represents a clinically realistic scenario, avoiding any prerequisites or data selection. Indeed, images are characterized by different quality levels (manually classified as bad, medium and high quality from clinicians) and representing different cardiac statuses (ejection fractions of these patients vary from 6% to 86%).

For each patient, two sequences were acquired showing the apical four-chamber and two-chamber views. According to the standard dimension criteria [17] (i.e., frames with the largest and lowest dimensions were set as ED and ES, respectively), ES and ED frames were determined. Each image was manually segmented into three regions, which were the left atrium (LA), left ventricular endocardium (LVendo) and left ventricular epicardium (LVepi). An annotation procedure was performed based on the opinions of three independent cardiologists. The masks created by the manual annotation procedure were considered ‘ground truth.’ Finally, 2000 echocardiographic images (500 patients by two chamber view by two frames) and the relative annotations were collected in the database.

Only 1800 images of 450 patients out of 500 were publicly available and were considered in this study. Then, this dataset was divided into training set (60%), validation set (10%) and testing set (30%), including 270 patients (1080 annotated images), 45 patients (180 annotated images) and 135 patients (540 annotated images), respectively.

### 3.2. Deep-Learning-Based Tool for Segmentation of Anatomical Structured of the Left Heart

**Figure 1:**
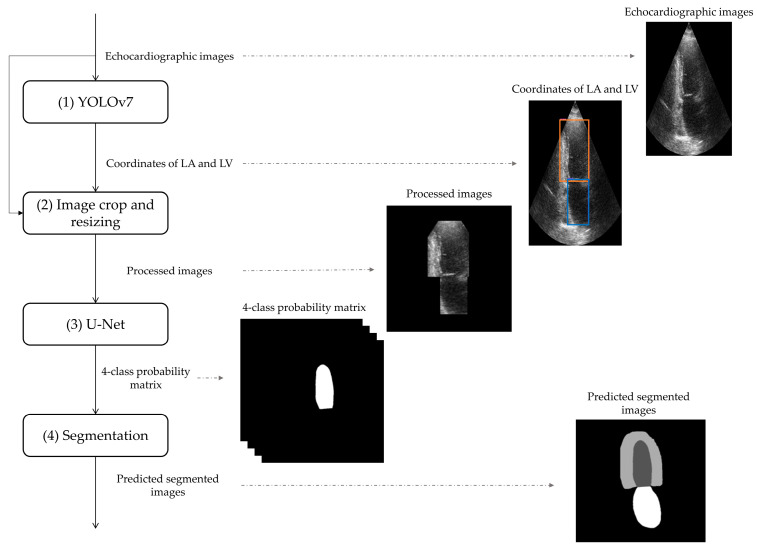
Block diagram of the proposed DL-based tool for segmentation of anatomical structures of the left heart by the use of echocardiographic images. Electrocardiographic images are initially analyzed by YOLOv7 (step 1) algorithm to identify the coordinates of the left atrium (LA, in blue) and of the left ventricle (LV, in orange). The coordinates of LA and LV are used to image cropping and resizing (step 2) and then, processed images are processed by U-Net (step 3). Finally, the obtained 4-class probability matrix is used to obtain the predicted segmented images by segmentation (step 4).

The proposed DL-based tool, represented in Figure 1, is composed of four steps: (1) the detection of the left heart by YOLOv7, (2) an image crop and resizing, (3) the U-Net application and (4) the segmentation of anatomical structures of the left heart. Its implementation was performed on Google Colab Pro, a cloud service allowing the possibility of selecting high system RAM (32 GB) and GPU hardware acceleration (NVIDIA Tesla P100 with 16 GB of video RAM) settings. Python language was used for all computation, by considering the Keras library built on TensorFlow backend.

#### 3.2.1. Detection of Anatomical Structures of the Left Heart by YOLOv7

The YOLO algorithm (i.e., You Only Look Once), was introduced in 2016 [21]. The main idea behind this algorithm was framing detection as a regression problem, so only one network is able to perform both predictions of the bounding box and its probability. YOLO works by dividing the input image into a grid of cells, which serves as the basis for predicting the presence and location of objects in the image; this makes it faster and more efficient than other object-detection algorithms that perform region-based processing; after predictions have been made for all cells, YOLO performs non-max suppression to eliminate redundant detections and return the most likely object detections.

The most recent version of YOLO is version 7. The authors of YOLOv7 implemented several structural modifications, such as the extended efficient layer aggregation network, model scaling techniques, re-parameterization planning and auxiliary head coarse-to-fine. All these modifications allowed YOLOv7 to overcome the previous versions, offering higher accuracy, faster performance, improved scalability, and greater flexibility for customization. YOLOv7 is free to use under GNU General Public License v3.0 license [22].

In this paper, we considered the free version of YOLOv7, which was trained on the training dataset with the aim of localizing the LA and LV from both four-chamber and two-chamber views. Input and output of YOLOv7 (Figure 1—step 1) are the echocardiographic images and the coordinates of LA and LV, respectively. The architecture of YOLOv7 was maintained unchanged; the initial learning rate and the number of epochs were set at 0.01 and 100, respectively.

#### 3.2.2. Image Crop and Resizing

Echocardiographic images have to be cropped to accord with the YOLOv7 output and resized in order to match the settings of the segmentation algorithm. Thus, the electrocardiographic images were cropped to accord with the coordinates of LA and LV and resized to 320 pixels × 320 pixels: if the image was bigger that normalized dimensions, it was resized by interpolation; otherwise, if the image was lower than normalized dimensions, it was zero-padded. The inputs of the image crop and resizing are the echocardiographic images and the coordinates of the region of interest, while the outputs are the processed images (Figure 1—step 2).

#### 3.2.3. U-Net Application

U-Net is a CNN whose architecture was designed for image segmentation tasks [20]. This architecture has a U-shape and, thus, it is composed of two paths, which are the encoder and the decoder. The encoder consists of multiple stages, and it has the aim of extracting high-level features; at each stage, the spatial resolution is decreased, and the number of channels is increased. The decoder also consists of multiple stages, but it has the aim of reconstructing the information derived by encoder; at each stage, the spatial resolution is increased, and the number of channels is decreased. The encoder path uses max pooling to decrease the spatial resolution while increasing the number of feature channels, and the decoder path uses transposed convolution layers to increase the spatial resolution while decreasing the number of feature channels. ‘Skip connections’ allow U-Net to combine low-level features from the early layers with high-level features from the later layers, which improves object localization and segmentation. Finally, the architecture includes a final layer that outputs a probability distribution over the classes for each pixel.

**Figure 2:**
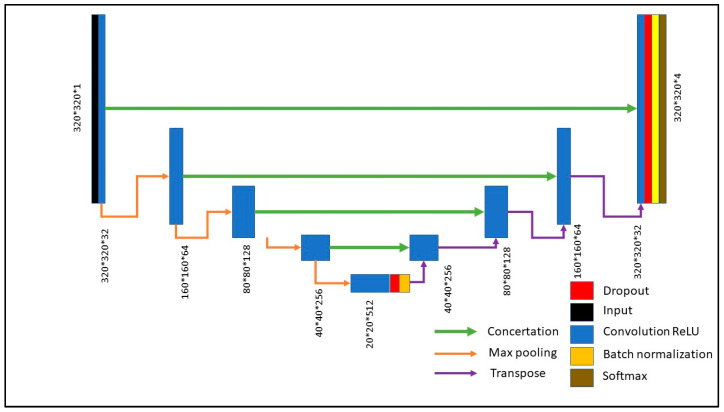
Architecture of proposed U-Net.

In this paper, the inputs of the U-Net were the processed images (Figure 1—step 3), having a size of 320 pixels × 320 pixels. The architecture of the proposed U-Net (Figure 2) was composed of an encoder composed of 5 stages, the feature map of which converged to 20 × 20 × 512, and a decoder composed of 5 stages and using transposed layers to perform up-sampling. The number of classes was set at 4, which are pixels belonging to LA, LVendo, LVepi and background. Supervised learning was applied, as well as the Dice coefficient (DCS) as loss function (Equation (1)):

(1) $$L_{\mathrm{DCS}}=1-\frac{1}{\sum_{k}\alpha_{k}k}\left[\sum_{k}\alpha_{k}k\frac{2\;\times \;\sum_{i\in I}u_{i}^{k}\mu_{i}^{k}}{\sum_{i\in I}u_{i}^{k}+\sum_{i\in I}\mu_{i}^{k}}\right]$$

where u is the predicted output of the network, μ is a one-hot encoding of the ground truth segmentation map, $\alpha_{k}$ is the weight associated to class k ∈ 1, 2, 3 (class related to background was ignored) being the pixel class. Adam was used as the optimization algorithm [23] (learning rate equal to 0.001, β_1_ equal to 0.9, β_2_ = 0.999, momentum equal to 0.99 and batch size equal to 10), and the number of epochs was set at 60. Values of all hyperparameters were empirically selected [24]. Outputs of the U-Net were a four-class matrix containing the probability of having a specific pixel in a specific class (Figure 1—step 3).

#### 3.2.4. Segmentation of Anatomical Structures of the Left Heart

U-Net provided a 4-class matrix containing the probability of having a specific pixel in a specific class. In order to obtain the predicted segmented images by segmentation (Figure 1—step 4), the pixels with the highest probability of belonging to LA, LVepi, LVendo or background were selected to be part of LA, LVepi, LVendo or background, respectively.

### 3.3. Evaluation Metrics

With the aim of evaluating the strength of the method, each image’s Dice similarity coefficients (DSC), Hausdorff’s distance (HD) and Jaccard index (JAC) were computed [25]. Calculation of all these evaluation metrics permitted a comparison between the LA, LVepi and LVendo of the predicted segmented images and the ground truth.

DSC and JAC for each i class can be defined as following (Equations (2) and (3)):

(2) $${DSC}_{i}=\frac{2\cdot TP}{2\cdot TP+FP+FN}$$

(3) $${JAC}_{i}=\frac{TP}{TP+FP+FN}$$

where TP are the true positives (pixels correctly classified in class i according to the ground truth), FP are the false positive (pixels wrongly classified in class i according to the ground truth) and TN are true negatives (pixels correctly not classified in class i according to the ground truth).

For each class, HD between the point P of the predicted class and the point GT of the ground truth class consisteds of the maximum of Euclidean distances, as shown in (Equation (4)):

(4) $$HD(P,GT)={max}_{P}\{{min}_{GT}\{||P,GT||\}\}$$

Considering the preprocessing of images (resizing), HD is represented in pixels. Distribution of DSC, JAC and HD of all patients are reported as mean value and standard deviations and classified according to the dataset (training, validation, or testing).

## 4. Results

**Figure 3:**
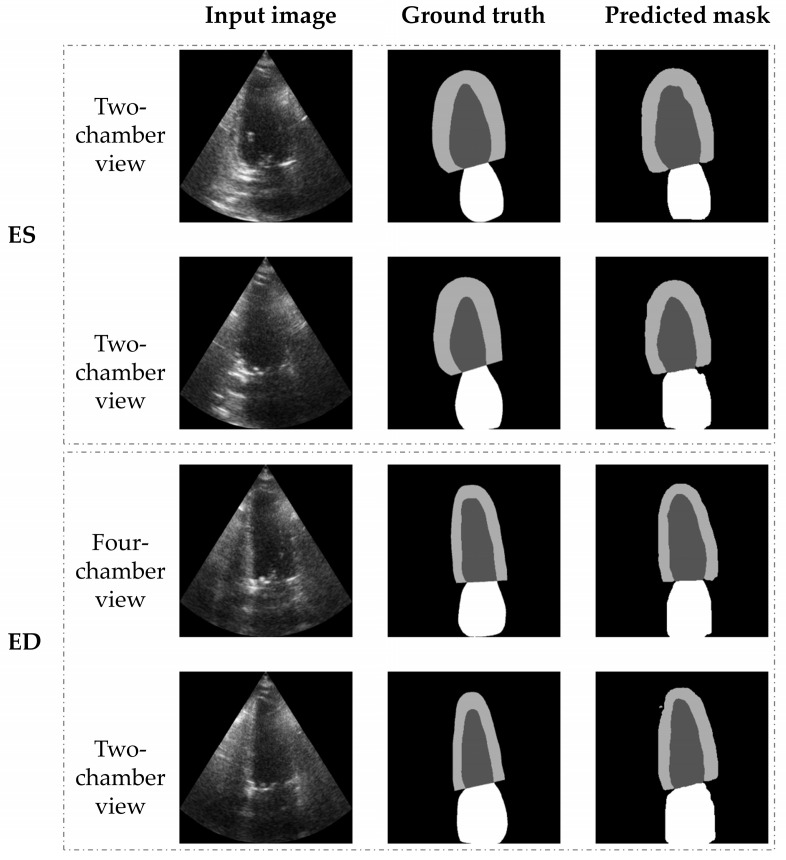
Example of cardiac segmentation into LA (white class), LVepi (light grey) and LVendo (dark grey).

Distributions of DSC, JAC and HD of all patients were classified according to the dataset (training, validation, or testing), and reported in Table 1. An example of cardiac segmentation into LA, LVepi and LVendo for both ES and ED is reported in Figure 3. Our DL-based tool provided very high results for cardiac segmentation of LA (DSC = 87.57%, JAC = 79.75% and HD = 4.07 pixels), LVepi (DSC = 85.59%, JAC = 75.38% and HD = 4.96 pixels) and LVendo (DSC = 92.63%, JAC = 86.76% and HD = 3.07 pixels). Despite the very high performance in all classes, the best recognized class was LVendo (DSC = 92.63 ± 6.60%, JAC = 86.76 ± 8.40% and HD = 3.81 ± 1.09 pixels).

## 5. Discussion

It is widely known that the segmentation of anatomical structures in the heart is an essential task, because the extracted features may be linked to cardiac dysfunctions, and thus clinically important for the detection of heart failure and infarction and the prediction of the occurrence of sudden cardiac death. Segmentation of the left ventricular structures is definitely clinically important. However, left atrium segmentation may further improve cardiac status evaluations and clinical diagnoses based on echocardiographic screening. Thus, differently from most of the works in the literature which simply identify the left ventricle, this study proposes a deep-learning-based tool able to segment the left ventricular endocardium, left ventricular epicardium and left atrium by combining an object-detection method, the YOLOv7 algorithm, with another convolutional neural network, U-Net. We selected the YOLOv7 algorithm because it guarantees high accuracy in combination with a low computational time. These properties make YOLOv7 a proper method for detecting anatomical structures from echocardiographic images. Indeed, echocardiographic tests are usually composed of different frames of a video, and the clinicians usually use this test to follow the cardiac movement. Thus, a fast detection algorithm may help the clinicians in real-time detection and evaluation of the anatomical structures of the left heart and, thus, of the global cardiac status. Moreover, combining detection and segmentation guarantees good performance even when training the tool using both two-chamber and four-chamber views at the same time, implying that there is no need to train two separate tools for each view.

Another advantage of our deep-learning-based tool is its high flexibility. Indeed, the tool showed reliable performance even though working with images characterized by different levels of quality (manually classified as bad, medium and high quality by clinicians), representing different cardiac statuses (ejection fractions of these patients varied from 6% to 86%). Considering the high complexity of our method, we decided to implement the deep-learning-based tool using cloud computing. This technology allows the method to be trained and tested in a machine-independent environment. Additionally, this design setting guarantees an easy reproducibility and integrability within any support-level of the pipeline. With this aim, we selected the Pro version of Google Colab, because it allows the selection of high RAM (34 GB) and GPU (NVIDIA Tesla P100—16 Gb video RAM) hardware acceleration settings. Despite the significant advantages provided by the Google Colab environment, our proposed method was not free of implementation challenges. Indeed, YOLOv7 and U-Net systems have 37,201,950 parameters and 8,544,548 parameters to be trained, respectively. Thus, the training computational time is around 8 h, when using the strong GPU of Google Colab. Moreover, our deep-learning-based tool considers a uniform size of chambers identified by YOLOv7 (Figure 1—Step 2), slightly limiting the information that U-Net may process. Thus, future studies will exploit novel solutions in order to speed up the training and may consider images with different sizes than the inputs of our U-Net.

In order to compare our deep-learning-based tool with the literature, we considered all the papers that performed a similar analysis and organized their contents in Table 2. If the work included more than one dataset, we reported only the results on the CAMUS dataset. Nevertheless, the comparison can be performed only qualitatively, due to the high variability of dataset, validation methods, included frames and chamber views, number and type of considered segmented anatomical structures, and evaluation metrics. In our study, we relied only upon the static data division of the CAMUS dataset. Other studies have used datasets (an echo-dynamic dataset [17] or a huge dataset of porcine images [11]) as training dataset and then the CAMUS dataset as testing dataset. This mixture of data makes the performance interpretation very difficult because training and testing data are not acquired in the same conditions. Three studies [9,10,14] have applied the cross-validation technique. Despite cross-validation being considered a robust validation method, it does not allow for the use of a unique model that can be inserted in the real clinical scenario. Distinct from all the other methods in the literature, we merged images of different views (two- and four-chamber views) and different frames (ES and ED). Indeed, when implementing the YOLOv7 as an object-detection algorithm before the segmentation, our deep-learning-based tool does not need a priori classification of views and frames, since it is able to manage all images together without the need of applying the same architecture to different image configurations and the provision of separate results. Finally, only two studies in the literature [13,14] focused on the segmentation of three cardiac anatomical structures. Despite their slightly higher performances, both studies selected the images a priori according to cardiac chamber views and did not apply an automatic cardiac structure identification. Thus, even though not providing the highest performance, our proposed deep-learning-based tool seems to be the best method in terms of generalization.

Ultimately, it is worthwhile to observe that our proposed method was designed to be clinically applicable. It considered annotations of three independent cardiologists as the gold standard, in order to minimize the well-known effects of inter-cardiologist variability and subjectivity. Indeed, we believe that final diagnostic decision regarding the segmentation of anatomical structures of the left heart should be taken by clinicians, and ultimately only supported by an automatic tool as ours. Future studies will definitely confirm the clinical usability of our deep-learning-based tool by collaborating with clinicians in real clinical scenarios.

## 6. Conclusions

Echocardiographic imaging of the left heart is an efficient and flexible tool which can be applied in clinical practice. Considering its performance here, future studies will focus on the implementation of a real-time version of the algorithm and on its usefulness for the estimation of important clinical indices, such as the ejection fraction.

## Figures and Tables

**Table 1:** Distribution of DSC, JAC and HD for all patients, classified according to the dataset (training, validation, or testing). The number of patients and images presented in each dataset are also reported.

			Training Dataset	Validation Dataset	Testing Dataset
Number of patients			270	45	135
Number of images			1080	180	540
Overall	LA	DSC (%)	95.12 ± 3.91	93.76 ± 7.36	87.57 ± 13.48
		JAC(%)	90.90 ± 5.41	88.86 ± 8.85	79.75 ± 15.76
		HD (pixels)	3.60 ± 0.83	3.68 ± 0.82	4.07 ± 1.08
	LVepi	DSC (%)	91.79 ± 2.47	89.08 ± 3.29	85.59 ± 7.14
		JAC(%)	84.93 ± 4.12	80.47 ± 5.25	75.38 ± 9.17
		HD (pixels)	4.32 ± 0.73	4.62 ± 0.81	4.96 ± 1.09
	LVendo	DSC (%)	95.18 ± 2.24	92.76 ± 4.64	92.63 ± 6.60
		JAC(%)	90.89 ± 3.90	86.81 ± 7.24	86.76 ± 8.40
		HD (pixels)	3.41 ± 0.81	3.66 ± 0.87	3.81 ± 1.09

**Table 2:** Comparison of our deep-learning-based tool with studies in the literature.

Ref.	Dataset (Patients/Images)	Dataset Split	View	Classes	Method	Performance (on CAMUS Dataset)		
						LA	LVepi	LVendo
[13]	CAMUS (406/1624)	10-fold cross-validation	Two and four chamber	LVendo and LVepi	U-Net	n.a.	ED: DSC = 95.4 ±2.3 HD = 6.0 ± 3.4 ES: DSC = 94.5 ±3.9 HD = 6.1 ±4.6	ED: DSC = 93.9 ± 4.3 HD = 5.3 ± 3.6 ES: DSC = 91.6 ±6.1 HD = 5.5 ±3.8
[14]	(1) CAMUS (500/n.a.) (2) custom dataset (137/n.a.)	5-fold cross-validation	Four chambers	LV	MFP-U-Net	n.a.	DSC = 95.3 ± 1.9 HD = 3.5 ± 0.9	
[15]	(1) custom dataset (8/1649) (2) CAMUS (450/1800)	n.a.	Two and four chambers	LVepi and LVendo	SegAN	n.a.	DSC = 85.9 ± 6.4 HD = 6.2 ± 1.2	DSC = 91.7 ± 7.1 HD = 5.1 ± 1.7
[16]	CAMUS (450/1800)	Static data division	Two and four chambers	LA, LVepi and LVendo	LFB-Net	Four-chamber view: DSC = 92.0 ± 4.0 HD = 5.2 ± 3.5 Two-chamber view: DSC = 92.0 ± 5.0 HD = 4.8 ± 2.8	Four-chamber view: DSC = 86.0 ± 6.0 HD = 6.7 ± 3.0 Two-chamber view: DSC = 88.0 ± 4.0 HD = 7.1 ± 3.9	Four-chamber view: DSC = 94.0 ± 3.0 HD = 5.0 ± 2.8 Two-chamber view: DSC = 94.0 ± 3.0 HD = 5.6 ± 3.2
[12]	(1) EchoNet-Dynamic (2500/5000) (2) CAMUS (500/2000)	Static data divison	Two and four chambers	LVepi and LVendo	PLANet	n.a.	ED: DSC = 96.2 ± 1.2 HD = 4.6 ± 1.5 ES: DSC = 95.6 ± 1.4 HD = 4.6 ± 1.4	ED: DSC = 95.1 ± 1.8 HD = 4.2 ± 1.4 ES: DSC = 93.1 ± 3.2 HD = 4.3 ± 1.5
[17]	CAMUS (450/1800)	5-fold cross-validation,	Two and four chambers	LA, LVepi and LVendo	Cardiac- SegNet	ED: DSC = 89.5 ± 8.5 HD = 2.2 ± 4.1 ES: DSC = 92.2 ± 5.5 HD = 2.7 ± 3.5	ED: DSC = 96.0 ± 1.6 HD = 2.9 ± 2.1 ES: DSC = 95.3 ± 2.2 HD = 2.8 ± 2.2	ED: DSC = 94.8 ± 2.4 HD = 2.3 ± 1.8 ES: DSC = 92.7 ± 4.3 HD = 2.3 ± 2.3
[18]	custom dataset (380/380)	Static data division	Four chambers	LV	Deep Res-U-Net	n.a.	ES: DSC = 82.1 ±0.8 JAC = 66.9 ± 6.4 HD = 23.8 ± 0.1 ED: DSC = 86.5 ± 1.1 JAC = 63.7 ± 9.6 HD = 19.7 ± 0.2	
[19]	(1) EchoNet-Dynamic (10,024/20,048) (2) CAMUS (400/800)	Static data division	Four chambers	LV	DeepLabV3	n.a.	DSC = 93.1 ± 0.04	
[20]	custom dataset	n.a.	n.a.	LVendo	YOLOv3 (Darknet53)	n.a.	n.a.	DSC = 93.6 ± 2.0 HD = 6.7 ± 1.8
This study	CAMUS (450/1800)	Static data division	Two and four chambers	LA, LVepi and LVendo	YOLOv7 and U-Net	DSC = 87.6± 13.5 JAC = 79.8 ± 15.8 HD = 4.1 ± 1.1	DSC = 85.6 ± 7.1 JAC = 75.4 ± 9.2 HD = 5.0 ± 1.1	DSC = 92.6 ± 6.6 JAC = 86.8 ± 8.4 HD = 3.8 ± 1.1

n.a.—not applicable.

## Data Availability

Data used for this study belong to CAMUS dataset [13], freely available to the link: https://www.creatis.insa-lyon.fr/Challenge/camus/, accessed on 3 September 2022.
